# Professionalism and non-technical skills in Radiology in the UK: a review of the national curriculum

**DOI:** 10.1186/s13104-018-3200-5

**Published:** 2018-02-05

**Authors:** F. Daley, D. Bister, S. Markless, P. Set

**Affiliations:** 1 Department of Radiology, Addenbrooke’s University Hospital, Addenbrooke’s, Cambridge University Hospital, Box 219, Hills Road, Cambridge, CB2 0QQ UK; 2 Guy’s Hospital, Great Maze Pond, London, SE1 9RT UK; 3 King’s Learning Institute, Waterloo Bridge Wing, Franklin-Wilkins Building, Waterloo Road, London, SE1 9NN UK

**Keywords:** Professionalism, Radiology, Postgraduate curriculum, Education, Patient Safety

## Abstract

**Objective:**

To drive quality and safe clinical practice, professional values and non-technical skills need to be explicit in all postgraduate medical curricula and appropriate assessment tools should be available for teachers to apply. We interrogate a national Radiology curriculum for content on professionalism and assessment tools, comparing it with the Royal College of Physicians’ 2005 document.

**Results:**

We found that whilst the knowledge for practising with professional values is embedded in the curriculum, the skills that have to be acquired have not been comprehensively developed. This is reflected in the restricted assessment tools that are mapped to each generic area. The terminology used in the Radiology curriculum was varied and the most frequently used descriptor for professionalism or behaviours pertaining to non-technical aspects was Good Medical Practice; a term used by our regulator, the General Medical Council, and to which our curriculum is mapped. If terminology is to be standardized in Britain collaboration with our regulator is key. We need standardized terminology to permit effective research and sharing of best practice. The Radiology curriculum encompasses all the values set out in the seminal document produced by the Royal College of Physicians in 2005, *Doctors in society: medical professionalism in a changing world.*

## Introduction

It is recognized that graduate medical education poses unique challenge for the delivery of safe patient care [1]. In this cohort distinctive features of errors in judgement and team-working led to 70% of malpractice claims [2]. In the UK, the cost of errors is significant [3]. Doctors work in teams and between professional disciplines non-technical skills (NTS) are paramount in enhancing safe practice. All trainees need generic skills of decision making, team-working, resource management and leadership [4]. Yet Greig et al. [5] found a lack of specific detail on learning objectives or assessment recommendation for NTS in many UK curricula.

Radiology is central to the management of patient care in modern medicine, it is a specialty which interacts with many specialties in primary and secondary care. The radiologist is increasingly part of the investigating team and problems often arise from poor communication rather than technical skill. There is scope for loss of information at every interaction and problems of this nature can lead to significant medical errors [6]. There is limited literature on professionalism or NTS in Radiology; that from North America reflects on definitions [7] and ‘how I do it’ [8] principles and cannot be easily translated to the UK given the major organizational differences between the two health care systems.

The wider literature on professionalism in medicine wrestles with definitions and the importance of practising with professional values with limited number of studies on how to implement [9] the teaching of these generic skills. Our study looks in depth at a national Radiology curriculum’s content for professionalism and identifies areas for curriculum development.

According to a seminal document produced by the Royal College of Physicians in 2005 (RCP 2005), *Doctors in society: medical professionalism in a changing world*, professionalism comprises a set of values, behaviours and relationships that underpin the trust the public has in doctors. This includes integrity, compassion, altruism, continuous improvement, excellence, and working in partnership with members of the wider healthcare team [10]. The King’s Fund *On being a doctor: redefining medical professionalism for better patient care*, suggests it is the ability to apply a body of specialist knowledge and skills along with a high degree of self-regulation and observation of explicit standards and ethical codes [11]. Definitions of professionalism are manifold, and while there have been many attempts to standardize a definition, there has been no universal agreement [12], one reason why it may be so difficult to place within a curriculum as reported by Greig et al. [5]. The values of professionalism are those soft skills sometimes termed non-technical skills. The anaesthetists are at the forefront of training in non-technical skills or aspects of professionalism [13].

If medical curricula were able to make specific professional values more explicit it will become easier to embed into clinical practice: trainees will be able to learn about them, practise them and improve on them in the workplace; teachers will be fully aware of them in order that they can act as mentors and assess their tutees.

To embed professionalism in the Radiology curriculum we need a clear definition. What it is to be professional, needs to be reflected in terms specific enough to permit assessment of the same.

We set out to interrogate the Royal College of Radiologists (RCR) curriculum [14] against the RCP 2005 document on professionalism because the values of professionalism defined in the latter underpins those recently termed NTS used by the anaesthetists [13].

## Main text

### Methods

The Royal College of Physicians’ 2005 document (RCP 2005), *Doctors in society: medical professionalism in a changing world*, sought to conceptualise medical professionalism and define it using a range of evidence and is considered to have done so very effectively by the regulator, General Medical Council (GMC).

We evaluate the following research questions:

1. Does the 2015 Radiology curriculum match up with the robust definition of professionalism set out in the RCP 2005 document?
2. Is it explicit in the behaviours that are required to be a radiologist?
3. What are the methods of assessments for professionalism?

Using qualitative case study methodology [15] in the context of UK Radiology training we examined the two important documents outlined above. The method used is document analysis to identify emerging themes in both documents [16]. The two documents were treated as data sets, which were read and re-read multiple times to identify the values of professionalism and the term ‘professionalism’ by a method called constant comparison method [17]. The data from the two large documents was analysed inductively for emerging themes, similarities and differences.

In addition, quantitative analysis was made for the descriptors of non-technical skills and values of professionalism as used in the current medical literature to triangulate with the qualitative inductive analysis [18] described previously.

### Results

#### Qualitative analysis

The Radiology curriculum is constantly updated, for the purpose of this study we used the most recent curriculum dated November 2015.

Compared to previous curricula the 2015 curriculum is clear and explicit about professionalism in Radiology training. In the introduction it sets out that ‘*satisfactory performance in professional practice will be expected throughout*..’ and that ‘*performance to be judged will be the basis of much of the assessments of generic skills and competences such as good medical practice, clinical care, professionalism and leadership*.’

In Section 2.4 Generic content under ‘*behaviours in the work place*’ professionalism is defined as ‘*to practise radiology employing values, behaviours and relationships that underpins the trust the public has in doctors and in accordance with the current GMC Good Medical Practice (GMP) guidance*’ [4]. This definition resonates with that provided by the RCP 2005 document and encompasses the GMC guidance.

The curriculum also defines the knowledge required for professionalism as ‘*concepts of modern medical professionalism, the relevance of professional bodies and when to seek support*’.

Skills of professionalism set out in the curriculum are the values defined in the RCP 2005 document of practising ‘*with integrity, compassion, altruism, continuous improvement, excellence, working in partnership with members of the wider health care team*’. The curriculum goes beyond these values and adds ‘*humility, insight, respect for cultural and ethnic diversity and regard for the principle of equity*’.

In professional behaviours the curriculum emphasizes the practice of patient-centred care, prudent and equitable use of healthcare resources. It is explicit that the trainee should practise with honesty and sensitivity, be able to cope with uncertainty and have the ability to accept and act positively on appropriate feedback. There is resonance with the RCP 2005 document on professional behaviours.

The curriculum expands and prescribes under three further sections on (i) working with colleagues, (ii) relations and communications with patients and (iii) personal qualities, the importance of explicit knowledge, skills and behaviours for training and assessments. Overarching themes of effective communication, development of clinical teams underpinned by the values of the medical profession as outlined in the RCP 2005 document and respect for patients and colleagues emerged.

Throughout the text of the document, the curriculum echoes the RCP 2005 document. It tells us that ‘*good communication is an essential component*’, we should have ‘*a sense of team*-*working within all spheres of practice*’ and ‘*a professional attitude to all aspects of clinical practice, which places good conduct at its centre*’. It goes further in saying we should be maintaining individual skills, knowledge and values throughout our career with ‘*a desire to commit to the dynamic nature of radiological practice’*.

There is clear explicit connection and mapping to the four domains of GMP throughout the curriculum.

#### Quantitative analysis

Of the 193 pages of curriculum 5 (2.6%) were dedicated to professionalism and non-technical skills in Radiology. We report on the number of times descriptors of professionalism or professional values appeared in the document within the brackets following each descriptor as follows. The words- professionalism (6), professional values (2), integrity (5), compassion (2), altruism (2), continuous improvement (2), humility (1), excellence (3), respect for others (2), ‘regard for principle of equity’ (3), insight (5), Good Medical Practice GMP (96), leadership (35), resource management (14), team working (6), decision making (8) situational awareness (0), human factors (1), non-technical skills (0) and reflective approach (27).

The quantitative analysis triangulated well with the qualitative analysis through multiple readings and inductive analysis for themes as described.

#### Assessments

The curriculum indicated that these professional behaviours were to be assessed by work-based assessments in the National Health Service (NHS) ePortfolio. There are five methods for assessment of professional or non-technical skills namely, mini-IPX (mini-imaging interpretation exercise), Rad-DOPS (Radiology-direct observation of procedural skills), MDTA (Multi-disciplinary team Assessment), MSF (multi source feedback) and Audit assessment.

The mini-IPX has 11 areas for assessment of which three are non-technical and they relate to

i. Interaction with patient/staff
ii. Judgement/insight
iii. Overall clinical judgement

The Rad-DOPS has 13 areas for assessment of which three are non-technical and they relate to

i. Communication with patient/staff
ii. Judgement/insight
iii. Explain procedure/risk/informed consent

The MDTA has eight areas of assessment of which five are non-technical and they relate to

i. Communication of information/ideas
ii. Collaborative approach/team working
iii. Time management/organizing effectively
iv. Self awareness
v. Leadership of team

The MSF has ten areas of assessment and all pertain to values of professionalism or NTS, these include communication, attitude, team working, reliability/punctuality, leadership, honesty and overall professional competence.

The Audit assessment can be used to demonstrate use of health care resource.

The mini-IPX and Rad-DOPS are elementary for assessment of professionalism or NTS, they are designed to assess technical skills with soft skills in the context of communicating with staff/patients.

The MSF is the only true assessment tool for values of professionalism and it is undertaken once a year.

### Discussion

Contrary to Greig’s et al. [5] findings we found that the RCR curriculum is explicit about what professionalism means in training and that the themes matched those of the RCP 2005 document. In addition the curriculum is mapped to the GMC guidance of good medical practice. This is a good starting point, however whilst the knowledge for practising with professional values is embedded in the curriculum the skills that have to be acquired have not been comprehensively developed. This is reflected in the restricted assessment tools that are mapped to each generic area. Unlike the assessment tools for anaesthetists in training [13] our tools are limited. More research is needed to refine assessment tools for non-technical skills in Radiology.

One study which attempted to make these values more explicit found that by outlining certain behaviours that are expected, and by motivating positive department behaviour with assessment by colleagues, patients and referring physicians, professionalism can be successfully implemented. These values ranged from the assessment of simple introductions to the ability to prioritize patients over other tasks, facilitating patient care by helping physicians with imaging and setting a realistic tone when talking to referring clinicians [8]. This is a significant finding for educators in developing and delivering the curriculum.

It is interesting to note that if we were to search for descriptors such as non-technical skills (0), situational awareness (0) or human factors (1) without multiple reading of the entire document we may not have come to the conclusion that the RCR curriculum is explicit in these skills. Fundamentally the descriptors for NTS are the values of professionalism. Another important finding is that the curriculum descriptor for professional values or NTS was ‘*good medical practice*’, this was the most frequently used term. We need to standardize terminology as suggested by Greig et al. [5]. NTS is more than professionalism and for progress to be made in assessment of the same, we propose that undergraduate and postgraduate medical curricula should be reviewed in light of recent literature. If educators can agree on standardized terminology this would make research and sharing of best practice more effective.

## Limitations

Our study is limited by the interrogation of the singular UK Radiology curriculum however the themes identified may be generalised to other curricula and used to inform further curricular development.

## Abbreviations

NHS: National Health Service
NTS: non-technical skills
RCR: Royal College of Radiologists
RCP: Royal College of Physicians
GMC: General Medical Council
UK: United Kingdom
GMP: Good Medical Practice
Rad-DOPS: Radiology-direct observation of procedural skills
MDTA: Multi-disciplinary team Assessment
MSF: multi source feedback
Mini-IPX: mini-imaging interpretation exercise
